# A214 CONSTRUCTION AND VALIDATION OF A FRAILTY INDEX FOR THE INFLAMMATORY BOWEL DISEASE POPULATION

**DOI:** 10.1093/jcag/gwae059.214

**Published:** 2025-02-10

**Authors:** N Willett, S Stewart, J Jones, O Theou, K Rockwood

**Affiliations:** Division of Digestive Care and Endoscopy, Nova Scotia Health Authority, Halifax, NS, Canada; Dalhousie University, Halifax, NS, Canada; Division of Digestive Care and Endoscopy, Nova Scotia Health Authority, Halifax, NS, Canada; Dalhousie University, Halifax, NS, Canada; Dalhousie University, Halifax, NS, Canada

## Abstract

**Background:**

Frailty can be defined as a state of vulnerability to adverse health events, resulting from a reduction in physiological processes that are protective for health. Measuring frailty in populations with chronic immune-mediated disease, such as Inflammatory Bowel Disease (IBD), is becoming increasingly important. A Frailty Index (FI) operates within the Deficits Accumulation model and is a valid and accepted method of measuring frailty. Much of the work done in the field of frailty and IBD describes the association of frailty with post-operative complications and mortality. These studies have used various methods to determine a patient’s degree of frailty such as use of ICD codes and claims-based scoring.

**Aims: Aims:**

To develop an FI for the IBD patient population using an accumulation of deficits approach.

**Methods:**

A standardized method for constructing an FI using data from the SPOR IMAGINE IBD cohort was applied to a pan-Canadian, observational prospective study. Seventy-seven deficits were selected to be incorporated into the index, most from psychological or gastrointestinal variables. Construct and content validity of the index was assessed. The index was trichotomized based on established cut points and was then compared to patient demographics in the baseline cohort.

**Results:**

An IBD Frailty Index (FI) was created using a validated 10 step process with baseline data from 2714 participants in the SPOR IMAGINE cohort. The IBD FI is made up of 77 items, and is primarily composed of physical, functional and psychological health deficits. The IBD FI showed a right skew, which is characteristic of most FIs. In this distribution the upper limit (99th percentile) of FI score is 0.529 displaying a submaximal limit, and the mean FI score was higher in women (0.19) (95% CI: 0.13, 0.15] compared to men (0.14), which are characteristic to valid FI tools. The mean age of our cohort was 45.8 years (SD: 15), mean FI score was 0.17 (SD: 0.12) (lower than in other immune mediated diseases), and 30.6% of our sample was categorized as frail (FI score >0.21). Surprisingly the IBD FI scores were not positively associated with age, though they were associated with IBD symptom severity (R=0.767).

**Conclusions:**

Frailty is an important health measure that provides a holistic picture of an individual’s health. We operationalized the accumulation of deficits approach to frailty and created a frailty measurement tool for the IBD population using variables from a wide variety of health domains. Future research projects using the IBD FI should investigate the scores’ predictive abilities for mortality and should explore the differences in frailty between patients diagnosed in middle of life (first epidemiological peak) versus later in life.

**Funding Agencies:**

None

